# Waterpipe smoking: Results from a population-based study in Qatar

**DOI:** 10.18332/tid/149907

**Published:** 2022-06-24

**Authors:** Ahmad AlMulla, Silva Kouyoumjian, Patrick Maisonneuve, Sohaila Cheema, Ravinder Mamtani

**Affiliations:** 1 Tobacco Control Center, WHO Collaborating Center for Treating Tobacco Dependence, Department of Medicine, Hamad Medical Corporation, Doha, Qatar; 2 Unit of Clinical Epidemiology, Division of Epidemiology and Biostatistics, European Institute of Oncology, Scientific Institute for Research, Hospitalization and Healthcare, Milan, Italy; 3 Institute for Population Health, Weill Cornell Medicine-Qatar, Doha, Qatar

**Keywords:** tobacco, smoking, waterpipe, prevalence, Qatar

## Abstract

**INTRODUCTION:**

Waterpipe smoking is common in the Eastern Mediterranean Region and is becoming more prevalent in Qatar. To better plan waterpipe smoking control strategies we aimed to: 1) determine the prevalence of waterpipe smoking and explore its patterns in Qatar; 2) describe the knowledge, attitudes, and practices related to smoking behaviors; 3) recognize locations of waterpipe smoking and symptoms experienced during waterpipe sessions; and 4) evaluate the frequency of waterpipe smoking and the initiation age.

**METHODS:**

We analyzed the data of a 7921 population-based survey of adults aged ≥18 years (nationals and expatriates), conducted in Qatar between March and December 2019. Out of 7105 surveys collected, 6904 were complete and included in the analysis.

**RESULTS:**

Of the 6904, 570 (8.3%; 95% CI: 7.7–9.0) were waterpipe tobacco smokers, 425 (10.6%) males and 145 (5.1%) females. The highest prevalence of waterpipe smoking was reported among people aged 18–24 years (10.6%). Of the 575 waterpipe smokers, 56.3% (n=324) were exclusive waterpipe smokers. Use of other tobacco products among waterpipe smokers was higher among Qataris (52.3%) than expatriates (37.7%). Waterpipe cafés were the most common location for waterpipe smoking, however, females preferred restaurants; 83.3% reported that waterpipe smoking is harmful, while 39.3% considered that it is less harmful than cigarette smoking.

**CONCLUSIONS:**

Waterpipe smoking prevalence is considerably high in Qatar, the second form of tobacco used. The formulation of new policies and enforcement of regulatory restrictions on waterpipe smoking are essential to reduce its uptake. Expansion in tobacco cessation services for women and poly-tobacco users is needed.

## INTRODUCTION

Waterpipe smoking, also called shisha, narghile, hubble-bubble, and hookah, consists of heated tobacco smoke inhalation after passing through water^1^. Waterpipe smoking is now increasingly popular globally^2^, and is one of the most common forms of tobacco smoked, particularly in the Eastern Mediterranean Region (EMR)^3,4^. According to GYTS school-based survey results, high prevalence of waterpipe smoking was reported among school students in the Gulf region^5^. Moreover, waterpipe smoking is the second common form of tobacco used among university students in Qatar^6^. However, in the United Arab Emirates, medwakh (traditional Arab pipe) smoking precedes waterpipe smoking^7^.The popularity of waterpipe smoking in the EMR is mostly due to the low price of tobacco in the region, weak tobacco control policies, and being a common habit in social gatherings^3,8^.

Most people incorrectly believe that waterpipe smoking is less harmful than cigarette smoking^9,10^. However, waterpipe smokers inhale large quantities of toxicants that may lead to tobacco-related disease^11-13^, including cancer^14^, and when compared to cigarette smokers, they can be exposed to an equal or greater health risk^15,16^. Toxic substances in tobacco smoke include carbon monoxide, nitric oxide, nicotine, polycyclic aromatic hydrocarbons, nanoparticles, volatile aldehydes, and others^1^. Waterpipe smoking is associated with an identified dependence effect exhibited by nicotine withdrawal symptoms trying to quit, similar to cigarette smoking^17-19^.

Despite its deleterious effects, waterpipe smoking is highly popular among university students, females, and in social gatherings^20-24^. Greater social acceptability and tolerance toward waterpipe smoking than cigarette smoking are some of the facilitating factors for female students, in some Arab countries, to use the waterpipe^25^. Nowadays, waterpipes are available for socializing at parties, in households, hotels, cafés, and in restaurants^10^. This trend for females is due to increased availability of appealing tobacco flavors, the flourishing café culture, affordability, and the absence of policies related to prevention of waterpipe smoking in contrast to the negative attitude toward cigarette smoking^2,26^.

The increase in waterpipe smoking has prompted the World Health Organization (WHO) to declare its use as a growing public health concern in its 2015 advisory note^27^. Within the WHO Convention on Tobacco Control (FCTC) framework, Qatar has adopted effective measures for tobacco control such as tax increase, smoke-free policies, ban on advertising and provision of tobacco intervention services along with free-of-cost complete cessation treatment^28,29^. Additionally, telephone-based smoking cessation counseling support during the COVID-19 pandemic has also been provided^30^. Though Qatar has signed and ratified the WHO Framework Convention on Tobacco Control (FCTC) in 2004, a high prevalence of cigarette use is reported in the country^31,32^ along with a growing number of waterpipe cafés that attract both adults and teenagers^33^. Qatar represents a culturally diverse society in the Middle East Region. Expatriates from over 100 nationalities and multiple ethnicities live in Qatar. According to a recent population-based study, the rate of overall tobacco use among adults in Qatar was 25.2%, with waterpipe being the second most common form of tobacco used^34^.

Due to the increasing practice and the potential harmful health effects of waterpipe smoking, and based on our previous published study about the epidemiology of tobacco use in Qatar^34^, we aimed to support the Framework of WHO Tobacco Free Initiative by: 1) determining the prevalence of waterpipe smoking and explore its patterns in Qatar; 2) describing the knowledge, attitudes, and practices related to smoking behaviors; 3) recognizing waterpipe smoking locations and symptoms experienced during waterpipe sessions; and 4) evaluating the frequency of waterpipe smoking and the initiation age. Therefore, this study focuses on waterpipe smoking in Qatar, which is not commonly assessed by previous studies. The study findings could be instructive in better planning waterpipe smoking control strategies in the country.

## METHODS

### Study design, population, and sampling

We used data from a nationwide, population-based cross-sectional study of adults aged ≥18 years (nationals and expatriates) conducted in Qatar from March to December 2019. Governmental employees and university students constituted the study reference population using multi-level cluster selection. The governmental employees were selected from the health sector, ministries and governmental offices, and the students were selected from four governmental universities to allow fair representation of Qatari adults. The subgroup information by regions was not sufficiently collected. Adjustment was not made for the effect of clustering during statistical analyses. However, the study’s research design was implemented based on a systematic process to ensure replicability. The final sample size was 7921, with an overall survey response rate of 89.7% (n=7105). Of the 7105 surveys collected, 6904 were complete and included in the analysis.

A self-administered country-adapted summarized version of the Global Adult Tobacco Survey (GATS) was developed. It was carefully administered with attention to protocol adherence by trained staff to guarantee a unified procedure. Study participants were provided with an envelope that included the survey along with an information sheet describing the study. To ensure that the anonymity and confidentiality of responses was preserved, the respondents were asked to complete the survey and return it via a sealed envelope.

The study protocol was approved by the Institutional Review Board, Medical Research Center, Hamad Medical Corporation, Qatar. The details of the methodology have been published previously^34^.

### Data collection

Data were collected using a self-administered questionnaire consisting of a country-adapted summarized version of the Global Adult Tobacco Survey (GATS). The study questionnaire included questions on key demographic characteristics, the use of all tobacco products including waterpipe smoking, cessation trials, location of waterpipe smoking, symptoms experienced during waterpipe smoking session, frequency of usage, age at initiation of waterpipe smoking, and knowledge, attitude and perceptions about smoking. In Qatar, waterpipe is commonly known as shisha and the two names will be used interchangeably in this article. People who are not of Qatari citizenship will be referred to as expatriates throughout the article.

### Sample size and data analysis

The sample size of the population-based survey study was 7921, with an overall survey response rate of 89.7%. Out of 7105 surveys collected, 6904 were complete and included in the analysis. Data analysis was performed using SPSS statistical software. Sample characteristics were expressed as frequency and percent (%) or mean ± standard deviation. The associations between the study variables were assessed using the chi-squared test, with statistical significance being defined as p≤0.05.

## RESULTS

### Prevalence of waterpipe smoking by sociodemographic characteristics

Out of the 6904 completed surveys, 575 (8.3%; 95% CI: 7.7–9.0) were waterpipe tobacco smokers. Table 1 shows the prevalence of waterpipe smoking according to sociodemographic characteristics. Of the total sample, 10.6% (n=425) of males and 5.1% (n=145) of females were current waterpipe smokers (p<0.001) (Table 1). Highest prevalence of waterpipe smoking was noted among people aged 18–24 years (10.6%) compared to the remaining age group categories (p<0.001). Current waterpipe smoking was higher among expatriates 9.2% (n=329) in comparison to Qataris 7.2% (n=7.2%; p=0.002), particularly among expatriates coming from the levant region in the Eastern Mediterranean Region (37.1%), namely from Jordan (13.4%), Palestine (12.8%), Syria (7.9%), and Lebanon (2.7%). Waterpipe smoking was highest among singles (9.5%) compared to married (7.6%) and divorced/widowed individuals (8.8%). No significant difference in waterpipe smoking was found between individuals of different educational level (p=0.177).

**Table 1:** Prevalence of waterpipe smoking according to sociodemographic characteristics, Qatar 2019

*Characteristics*	*Respondents in the survey n (%)^a^*	*Waterpipe smokers n (%)^a^*	Prevalence of waterpipe smoking % (95% CI)*	*p*
**Total**	6904 (100)	575 (100)	8.3 (7.7–9.0)	
**Sex**				
Male	4002 (58.4)	425 (74.6)	10.6 (9.7–11.6)	<0.001
Female	2854 (41.6)	145 (25.4)	5.1 (4.3–6.0)	
**Age** (years)				
18–24	1351 (22.5)	143 (27.8)	10.6 (8.9–12.2)	<0.001
25–34	1882 (31.4)	177 (34.4)	9.4 (8.1–10.7)	
35–44	1692 (28.2)	132 (25.6)	7.8 (6.5–9.1)	
45–54	811 (13.5)	50 (9.7)	6.2 (4.5–7.8)	
≥55	266 (4.4)	13 (2.5)	4.9 (2.3–7.5)	
**Nationality**				
Qatari	3263 (47.6)	235 (41.7)	7.2 (6.3– 8.1)	0.002
Expatriate	3585 (52.4)	329 (58.3)	9.2 (8.2–10.1)	
**Expatriates region of origin^b^**				
**Eastern Mediterranean Region (EMR)**	1835 (51.2)	230 (69.0)	12.5 (11.0–14.0)	
*Levant region*	567 (15.8)	122 (37.1)	21.5 (18.1–24.9)	
Jordan	242 (6.8)	44 (13.4)	18.2 (13.3–23.0)	
Syria	108 (3.0)	26 (7.9)	24.1 (16.0–32.1)	
Palestine	152 (4.2)	42 (12.8)	27.6 (20.5–34.7)	
Lebanon	61 (1.7)	9 (2.7)	14.8 (5.9–23.7)	
Other (Turkey)	4 (0.1)	1 (0.3)	-	
*Other EMR regions*	1268 (35.4)	108 (32.8)	8.5 (7.0–10.1)	
Egypt	524 (14.6)	54 (16.4)	10.3 (7.7–12.9)	
Sudan	245 (6.8)	22 (6.7)	9.0 (5.4–12.6)	
Pakistan	143 (4.0)	11 (3.3)	7.7 (3.3–12.1)	
Tunisia	95 (2.6)	5 (1.5)	5.3 (0.8 – 9.8)	
Yemen	86 (2.4)	7 (2.1)	8.1 (2.4–13.9)	
Other^c^	175 (4.9)	9 (2.7)	5.1 (1.9–8.4)	
**Region of the Americas (AMR)**	88 (2.5)	12 (3.6)	13.6 (6.5–20.8)	
United States	45 (1.3)	5 (1.5)	11.1 (1.9–20.3)	
Canada	37 (1.0)	6 (1.8)	16.2 (4.3–28.1)	
Other^d^	6 (0.2)	1 (0.3)	-	
**European Region (EUR)**	115 (3.2)	5 (1.5)	4.3 (0.6–8.1)	
United Kingdom	66 (1.8)	4 (1.2)	6.1 (0.3–11.8)	
Other^e^	49 (1.4)	1 (0.3)	-	
**South-East Asian Region (SEAR)**	581 (16.2)	17 (5.2)	2.9 (1.6–4.3)	
India	533 (14.9)	15 (4.6)	2.8 (1.4–4.2)	
Other^f^	48 (1.3)	2 (0.6)	-	
**Western Pacific Region**	170 (4.7)	5 (1.5)	2.9 (0.4–5.5)	
Philippines	140 (3.9)	4 (1.2)	2.9 (0.1–5.6)	
Other^g^	30 (0.8)	1 (0.3)	-	
**African Region^h^**	30 (0.8)	4 (1.2)	13.3 (1.2–25.5)	
Unknown	766 (21.4)	56 (17.0)	7.3 (5.5–9.2)	
**Marital status**				
Single	2555 (37.4)	243 (42.7)	9.5 (8.4–10.6)	0.019
Married	4072 (59.6)	308 (54.1)	7.6 (6.8–8.4)	
Divorced/widowed	205 (3.0)	18 (3.2)	8.8 (4.9–12.7)	
**Education level**				
Secondary	1418 (20.8)	106 (18.8)	7.5 (6.1–8.8)	0.177
University	4240 (62.1)	371 (65.7)	8.8 (7.9–9.6)	
Postgraduate	1167 (17.1)	88 (15.6)	7.5 (6.0–9.1)	

a The numbers may not total for some variables due to missing values.

b Data related to expatriates’ nationality is presented by WHO region categorization, and EMR region is further divided into Levant region that includes Syria, Lebanon, Jordan, Israel (none in our sample), Palestine and Turkey and other EMR region.

c Afghanistan, Bahrain, Oman, United Arab Emirates, Djibouti, Iran, Iraq, Kuwait, Libya, Morocco, Oman, Saudi Arabia, and Somalia.

d Brazil, Dominica, Mexico, and Venezuela.

e Albania, Poland, Bosnia, Denmark, Netherlands, France, Germany, Greece, Ireland, Italy, Kosovo, Lithuania, Portugal, Romania, Russia, Spain, Switzerland, Sweden, and Ukraine.

f Bangladesh, Indonesia, Nepal, and Sri Lanka.

g Australia, New Zealand, and Malaysia.

h Burkina Faso, Eritrea, Ethiopia, Gambia, Ghana, Kenya, Mali, Mauritania, Nigeria, Rwanda, South Africa, and Zimbabwe.

* Prevalence and confidence interval of waterpipe smoking were not calculated for some of the ‘Other’ categories due to small numbers.

### Use of other tobacco products

Details about the number and percentage of other tobacco products used among waterpipe smokers are found in Table 2. Out of the total waterpipe smokers (n=575), 56.3% exclusively smoked waterpipe (n=324) and the remaining 43.7% (n=251) concomitantly used other types of tobacco; 76.6% (n=111) of females and 49.6% (n=211) of males were exclusive waterpipe smokers. Exclusive waterpipe smoking was higher among expatriates (62.3%) than Qataris (47.7%). The numbers of different combinations of waterpipe smoking are not exclusive, the same subject may be listed in more than one category. More than three-fifths of the sample (62.5%) used only one type of tobacco product with waterpipe smoking. However, almost half of Qataris (44.8%) used two or more tobacco products with waterpipe smoking. The combination of cigarette with waterpipe smoking concomitant with other forms of tobacco was the most prevalent combination reported (80.5%), followed by combinations of e-cigarettes (23.5%) and medwakh (23.1%), except among Qataris where medwakh preceded e-cigarette use.

**Table 2:** Use of other tobacco products and cessation efforts in waterpipe smokers by sex and nationality, Qatar 2019

	*Total n (%)^a^*	*Male n (%)*	*Female n (%)*	*p*	*Qatari n (%)*	*Expatriate n (%)*	*p*
	575 (100)	425 (100)	145 (100)		235 (100)	329 (100)	
**Waterpipe smoking**							
Exclusive	324 (56.3)	211 (49.6)	111 (76.6)	<0.001	112 (47.7)	205 (62.3)	<0.001
Concomitant	251 (43.7)	214 (50.4)	34 (23.4)		123 (52.3)	124 (37.7)	
**Number of tobacco types**							
1	157 (62.5)	130 (60.7)	24 (70.6)	0.427	68 (55.3)	85 (68.5)	0.040
2	65 (25.9)	57 (26.6)	8 (23.5)		35 (28.5)	30 (24.2)	
≥3	29 (11.6)	27 (12.6)	2 (5.9)		20 (16.3)	9 (7.3)	
**Most common types^b^**							
Cigarettes	202 (80.5)	170 (79.4)	29 (85.2)	0.831	99 (80.5)	100 (80.6)	0.091
Medwakh (traditional Arab pipe)	58 (23.1)	55 (25.7)	3 (8.8)		36 (29.3)	22 (17.7)	
Cigar	26 (10.4)	23 (10.7)	3 (8.8)		10 (8.1)	16 (12.9)	
Smokeless	29 (11.6)	27 (12.6)	2 (5.9)		25 (20.3)	4 (3.2)	
E-cigarettes	59 (23.5)	51 (23.8)	8 (23.5)		31 (25.2)	27 (21.8)	
Heated tobacco product (HTP)	11 (4.4)	9 (4.2)	2 (5.9)		5 (4.1)	6 (4.8)	

a The numbers may not total 575 for some variables due to missing values.

b The same subject may be listed in more than one category.

### Smoking knowledge, attitude, and perceptions among waterpipe smokers

Table 3 shows details regarding smoking knowledge, attitude, and perceptions among waterpipe smokers stratified by sex and nationality. Most participants (85.3%) agreed that smoking tobacco caused serious illness (84.3% males and 88.2% females; 80.4% Qataris and 88.6% expatriates). One-fifth of waterpipe smokers (20.0%) did not agree or were not aware that breathing tobacco smoke while other people smoked can cause serious illness among non-smokers.

**Table 3:** Smoking knowledge, attitude and perceptions by sex and among Qatari and expatriate waterpipe smokers, 2019 (N=575)

*Characteristics*	*Total n (%)^a^*	*Male n (%)*	*Female n (%)*	*p*	*Qatari n (%)*	*Expatriate n (%)*	*p*
Total	575 (100)	425 (100)	145 (100)		235 (100)	329 (100)	
**Smoking tobacco causes serious illness**							
Yes	464 (85.3)	344 (84.3)	120 (88.2)	0.279	180 (80.4)	281 (88.6)	0.028
No	44 (8.1)	33 (8.1)	11 (8.1)		24 (10.7)	20 (6.3)	
Does not know	36 (6.6)	31 (7.6)	5 (3.7)		20 ( 8.9)	16 (5.0)	
**Breathing other people’s smoke causes serious illness in non-smokers**							
Yes	433 (80.0)	321 (79.9)	112 (80.6)	0.451	178 (80.9)	252 (79.5)	0.265
No	41 (7.6)	28 (7.0)	13 (9.4)		20 (9.1)	21 (6.6)	
Does not know	67 (12.4)	53 (13.2)	14 (10.1)		22 (10.0)	44 (13.9)	
**Smoking tobacco causes stroke**							
Yes	230 (42.2)	172 (42.3)	58 (42.0)	0.690	92 (41.1)	138 (43.4)	0.490
No	123 (22.6)	95 (23.3)	28 (20.3)		57 (25.4)	67 (21.1)	
Does not know	192 (35.2)	140 (34.4)	52 (37.7)		75 (33.5)	113 (35.5)	
**Smoking tobacco causes heart attack**							
Yes	375 (69.2)	272 (67.2)	103 (75.2)	0.193	155 (69.2)	218 (69.0)	0.167
No	60 (11.1)	49 (12.1)	11 (8.0)		31 (13.8)	30 (9.5)	
Does not know	107 (19.7)	84 (20.7)	23 (16.8)		38 (17.0)	68 (21.5)	
**Smoking tobacco causes lung cancer**							
Yes	473 (86.9)	345 (85.0)	128 (92.8)	0.058	187 (84.2)	284 (89.0)	0.057
No	27 (5.0)	24 (5.9)	3 (2.2)		17 (7.7)	10 (3.1)	
Does not know	44 (8.1)	37 (9.1)	7 (5.1)		18 (8.1)	25 (7.8)	
**Waterpipe smoking is harmful**							
Yes	453 (83.3)	339 (83.3)	114 (83.2)	0.501	181 (81.2)	269 (84.9)	0.222
No	58 (10.7)	41 (10.1)	17 (12.4)		30 (13.5)	28 (8.8)	
Does not know	33 (6.1)	27 (6.6)	6 (4.4)		12 (5.4)	20 (6.3)	
**Waterpipe smoking is less harmful than cigarette smoking**							
Yes	215 (39.3)	163 (39.9)	52 (37.7)	0.343	95 (42.2)	121 (38.1)	0.167
No	269 (49.2)	195 (47.7)	74 (53.6)		111 (49.3)	154 (48.4)	
Does not know	63 (11.5)	51 (12.5)	12 (8.7)		19 (8.4)	43 (13.5)	
**Think or believe that most smokers can stop if they want to**							
Yes	409 (75.5)	308 (76.2)	101 (73.2)	0.493	162 (72.6)	242 (77.1)	0.486
No	89 (16.4)	62 (15.3)	27 (19.6)		42 (18.8)	48 (15.3)	
Does not know	44 (8.1)	34 (8.4)	10 (7.2)		19 (8.5)	24 (7.6)	
**Recommend waterpipe to your friends or to other people**							
Yes	92 (16.9)	65 (16.0)	27 (19.7)	0.318	42 (18.7)	49 (15.6)	0.349
No	51 (83.1)	341 (84.0)	110 (80.3)		183 (81.3)	265 (84.4)	

a The numbers may not total 575 for some variables due to missing values.

Less than half of the sample (42.2%) reported that smoking tobacco can cause stroke, whereas 69.2% reported that smoking tobacco can cause a heart attack, and 86.9% reported that smoking tobacco can cause lung cancer. Interestingly, 83.3% reported that waterpipe smoking is harmful. However, 39.3% confirmed that waterpipe smoking is less harmful than cigarette smoking with comparable proportion among males (39.9%), females (37.7%), Qataris (42.2%) and expatriates (38.1%). Moreover, 75.5% reported that they think or believe that most smokers can stop if they want to. Surprisingly, 16.9% recommend waterpipe to their friends or to other people, and this was slightly higher among females (19.7%) than males (16.0%), however not significant p=0.318, and also higher among Qataris (18.7%) than expatriates (15.6%), p=0.349.

### Cessation efforts in waterpipe smokers

**Figure 1:**
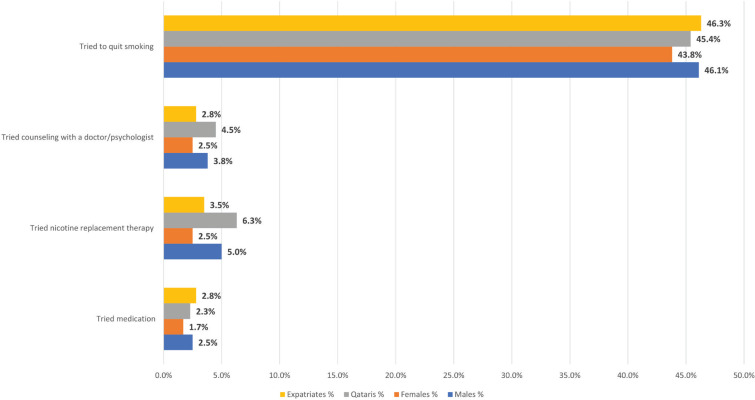
Cessation efforts in waterpipe smokers, Qatar 2019

Figure 1 shows the percentage of waterpipe smokers’ cessation trials by sex and nationality. Almost half of waterpipe smokers (n=475) tried to quit smoking during the past 12 months (45.5%). Compared to Qataris, expatriates tried to quit more (45.4% vs 46.3%) and females tried to quit less than males (43.8% vs 46.1%). Nevertheless, only 2.3% of the study population tried to use prescription medication, 4.5% tried to use nicotine replacement therapy, and 3.5% tried to use counseling with a physician/psychologist to stop smoking during the past 12 months.

### Waterpipe locations and symptoms experienced during waterpipe smoking sessions

Different aspects of waterpipe smoking by selected demographic characteristics are found in Table 4 such as locations of waterpipe smoking and symptoms experienced during waterpipe sessions, dependence on waterpipe smoking, and age of initiation. The most common waterpipe smoking locations were waterpipe cafés (65.3%), followed by restaurants (41.5%), and homes (31.4%) among both Qataris and expatriates, and by the different age groups. However, the majority of males frequented waterpipe cafés (69.8%), while the majority of females frequented restaurants (60.7%) for their waterpipe smoking session.

**Table 4:** Aspects of waterpipe smoking by selected demographic characteristics, Qatar 2019

	*Total*	*Sex*		*Missing*	*Nationality*		*Missing*	*Age (years)*					*Missing*
		*Male*	*Female*		*Qatari*	*Expatriate*		*18–24*	*25–34*	*35–44*	*45–54*	*≥55*	
**Location^a^**	516 (100)	391 (100)	122 (100)	3 (100)	210 (100)	297 (100)	9 (100)	132 (100)	155 (100)	122 (100)	48 (100)	12 (100)	47 (100)
Restaurant	214 (41.5)	139 (35.5)	74 (60.7)	1	73 (34.8)	136 (45.8)	5	57 (43.2)	75 (48.4)	45 (36.9)	11 (22.9)	5 (41.7)	21
WP cafe	337 (65.3)	273 (69.8)	61 (50.0)	3	149 (71.0)	185 (62.3)	3	103 (78.0)	97 (62.6)	67 (54.9)	29 (60.4)	8 (66.7)	33
Home	162 (31.4)	104 (26.6)	58 (47.5)	-	41 (19.5)	114 (38.4)	7	38 (28.8)	51 (32.9)	36 (29.5)	17 (35.4)	4 (33.3)	16
Other	36 (7.0)	33 (8.4)	3 (0.2)	-	22 (10.5)	14 (4.7)	-	8 (6.1)	9 (5.8)	12 (9.8)	5 (104)	1 (8.3)	1
**Symptoms^a^**	348 (100)	244 (100)	100 (100)	4 (100)	138 (100)	200 (100)	10 (100)	96 (100)	104 (100)	78 (100)	25 (100)	6 (100)	39 (100)
Headache	97 (27.9)	57 (23.4)	39 (39)	1	37 (26.8)	56 (28.0)	4	32 (33.3)	25 (24.0)	21 (26.9)	6 (24)	1 (16.7)	12
Dizziness	179 (51.4)	114 (46.7)	63 (63)	2	66 (47.8)	109 (54.5)	4	50 (52.1)	59 (56.7)	39 (50)	12 (48)	1 (16.7)	18
Blurred vision	18 (5.2)	13 (5.3)	5 (5)	-	11 (8.0)	7 (3.5)	-	4 (4.2)	5 (4.8)	2 (2.6)	3 (12)	0 (0)	4
Cough	60 (17.2)	46 (18.9)	14 (14)	-	17 (12.3)	40 (20.0)	3	11 (11.5)	19 (18.3)	18 (23.1)	5 (20)	1 (16.7)	6
Palpitation	69 (19.8)	38 (15.6)	31 (31)	-	26 (18.8)	42 (21.0)	1	16 (16.7)	25 (24.0)	17 (21.8)	4 (16)	2 (33.3)	5
Other	45 (12.9)	38 (15.6)	6 (6)	1	20 (14.5)	24 (12.0)	1	9 (9.4)	12 (11.5)	14 (17.9)	1 (4)	2 (33.3)	7
**Frequency of WP/week**	464 (100)	343 (100)	116 (100)	5 (100)	188 (100)	269 (100)	7 (100)	112 (100)	141 (100)	118 (100)	42 (100)	8 (100)	43 (100)
Average	4.7±6.4	5.1±7.1	3.4±3.9	-	5.5±5.7	4.0±6.7	-	2.9±4.2	4.4±4.9	5.1±8.3	7.9±8.3	4.8±2.7	-
≤1	179 (38.6)	127 (37.0)	51 (44.0)	1	52 (27.7)	125 (46.5)	2	58 (51.8)	59 (41.8)	39 (33.1)	10 (23.8)	2 (25.0)	11
2–4	131 (28.2)	94 (27.4)	37 (31.9)	-	53 (28.2)	77 (28.6)	1	36 (32.1)	32 (22.7)	42 (35.6)	7 (16.%)	1 (12.5)	13
≥5	154 (33.2)	122 (35.6)	28 (24.1)	4	83 (44.1)	67 (24.9)	4	18 (16.1)	50 (35.5)	37 (31.4)	25 (595)	5 (62.5)	19
**Initiation age** (years)	524 (100)	395 (100)	125 (100)	4 (100)	221 (100)	296 (100)	7 (100)	132 (100)	164 (100)	119 (100)	48 (100)	11 (100)	50 (100)
Average	20.6±6.0	20.0±6.0	22.4±5.8	-	19.6±5.6	21.3±6.2	-	16.4±2.6	20.8±4.3	22.0±5.8	25.5±8.6	21.9±6.2	-
≤15	91 (17.4)	83 (21.0)	6 (4.8)	2	53 (24.0)	38 (12.8)	-	43 (32.6)	18 (11.0)	13 (10.9)	4 (8.3)	3 (27.3)	10
16–18	149 (28.4)	116 (29.4)	33 (26.4)	-	62 (28.1)	85 (28.7)	2	67 (50.8)	34 (20.7)	30 (25.2)	9 (18.8)	1 (9.1)	8
>18	284 (54.2)	196 (49.6)	86 (68.8)	2	106 (48.0)	173 (58.4)	5	22 (16.7)	112 (68.3)	76 (63.9)	35 (72.9)	7 (63.6)	32

a The same subject may be listed in more than one category. Numbers are given as frequency and percent (%), or mean ± standard deviation. WP: waterpipe.

The main symptoms experienced by waterpipe smokers were dizziness (51.4%), headache (27.9%), palpitations (19.8%), cough (17.2%), and blurred vision (5.2%) (Table 3). When comparing by nationality the reported symptoms were similar. However, on comparing by sex and by age groups, the commonest symptoms reported by males aged 35–44 years and 45–54 years were different as in descending order: dizziness, headache, cough, palpitations, blurred vision and others.

### Frequency of usage and age of initiation of waterpipe smoking

Table 4 shows frequency of usage of waterpipe smoking and age of initiation. The overall mean ± SD number of waterpipes smoked per week was 4.7±6.4 (Qataris 5.5±5.7 and expatriates 4.0±6.7). The average number of waterpipes smoked per week among females was lower compared to males (3.4±3.9 vs 5.1±7.1). The mean number of waterpipes smoked per week increased with increase in each age group category reaching up to 7.9±8.3 among those aged 45–54 years. Overall, 38.6% smoked waterpipe ≤1/week. However, 44.1% of Qataris smoked waterpipe ≥5/week and 46.5% of expatriates smoked ≤1/week. More than half of people aged 18–24 years smoked ≤1 waterpipe per week, yet 59.5% of people aged 45–54 years smoked ≥5 waterpipes/week (Table 4).

The mean age ± SD of starting waterpipe smoking was 20.6±6.0 years (males 20.0±6.0 and females 22.4±5.8). For Qataris, it was 19.6±5.6 years, lower compared to expatriates 21.3±6.2 years, and lower than the average stated above. Overall, 45.8% reported smoking waterpipe before the age of 18 years. More than half of males (50.4%) reported smoking initiation before 18 years, however, 68.8% of females reported smoking after 18 years. For the people aged 18–24 years, half of them started smoking between the age of 16 and 18 years (50.8%) (Table 4).

## DISCUSSION

Of the total sample, the prevalence of waterpipe smoking among adults ≥18 years in Qatar is 8.3% (95% CI: 7.7–9.0), higher than the overall rate of Global Adult Tobacco Survey (GATS) in 2013, which reported that 3.4% of people aged ≥15 years smoked waterpipe^35^. In the WHO EMR, waterpipe smoking is second only to cigarette smoking and, in some countries, has surpassed cigarette use like in Lebanon^2,3^. Cigarette and waterpipe smoking is a significant health threat among university students in Egypt, Kuwait, Kingdom of Saudi Arabia, Jordan, Libya, Lebanon, Yemen, Palestine and United Arab Emirates^4^. Similarly, our results show that the highest proportion of waterpipe smokers are aged 18–24 years. This supports previous studies reporting that young adults are more likely to engage in waterpipe tobacco use^3,4^.

The prevalence of waterpipe smoking among males was twice the prevalence among females, however in a previously published study, there was a big difference of tobacco use among males and females (36.6% and 9.2%). This could be largely explained by the social appeal and the cultural tolerance of females smoking waterpipe and not cigarettes in traditional societies^36^. Females in the region tend to underreport their smoking behavior^37^, however this may not be a major problem in our study since the survey was self-administered and the completed surveys were returned in a sealed envelope to ensure optimum privacy and confidentiality. Though results are not shown, female waterpipe smokers were predominantly expatriates (16.1% Qataris vs 83.9% expatriates). The Qatari society tends to be conservative in most of its aspects because of its traditional and cultural influences, where females are highly unlikely to smoke. The implemented policy framework is the same for the whole population of Qatar, where tobacco use is not encouraged and preserving overall health is highly promoted. Not surprisingly, high prevalence of waterpipe smoking was reported among expatriates coming from the Levant countries due to cultural similarities where waterpipe smoking is a socially acceptable behavior^3,20^ and poorly regulated^26^.

Being single involves fewer social responsibilities and less financial stress that prevents smoking^38^, however this was not true for our sample, where waterpipe smoking was highest among unmarried individuals. These individuals may experience social isolation and not having spousal support which could be a trigger for unhealthy lifestyles or behaviors. Also, within the prevailing culture that highly respects family values, it is possible that married individuals are likely to abstain from smoking in front of the children and family.

Studies show that waterpipe smoking is usually not a daily practice and many regular smokers usually use more than one type of tobacco^2,9^. Waterpipe smokers are at risk of initiation of poly-tobacco use and are highly likely to become cigarette smokers in the future1. In fact, waterpipe smoking is a possible risk factor for other forms of tobacco consumption, including electronic cigarettes^39^. Data from 2013 GATS in Qatar show that current e-cigarette use among those aware of e-cigarettes was 1.8%^40^. In another recent study, exclusive electronic cigarette use was reported as 2.0% in Qatar, however concomitant with other types of tobacco products, it reached up to 11.3%^34^. Dual usage may promote and reinforce nicotine addiction. For instance, in a study conducted in Jordan, dual users were associated with higher waterpipe nicotine dependence compared to only waterpipe smokers^41^, which may postpone tobacco cessation and lower chances of successful cessation^42^. Therefore, dual and poly-tobacco use is an emerging pattern^2^ that is becoming a public health concern since their synergistic health effects remain mostly unknown. Our study findings can be used to inform new policy recommendations and to enact new regulatory laws and restrictions that do not apply to electronic cigarettes and to other tobacco forms. Moreover, they can aid in the design of cessation interventions relevant to the unprecedented increase in dual and multiple tobacco product use, especially among the Qatari nationals where medwakh use precedes e-cigarette use.

Importantly, this study identified that females tried less to quit smoking than males during the past 12 months. This might be attributed to the misconception that a lesser risk is associated compared with cigarette smoking, which possibly gives them a feeling of security regarding waterpipe smoking^10^. Future studies can explore factors that are barriers for treatment such as stigma, lack of education, and knowledge. Female-specific tobacco programs and services tailored to meet their preferences may reduce these barriers and motivate them to initiate treatment^43^. However, for males who tried to quit, their uptake of medication to stop smoking including nicotine replacement therapy and counseling was very low. Qatar is currently well placed to help tobacco smokers to quit, with the expansion of tobacco dependence treatment services across Qatar along with coverage for full cost of tobacco cessation support^29^. Yet, marketing of such smoking cessation treatments must be improved in the country.

In our study, waterpipe cafés were the most common place of smoking. Although smoke-free laws for clean indoor air exist in Qatar, waterpipe cafés are frequently exempted^33^. This is true for most countries where they do not address and do not effectively implement waterpipe tobacco smoking regulations and legislations^44^. However, most females in our study preferred restaurants for waterpipe smoking. This may be due to its popularity, accessibility and convenience and the availability of a variety of choices in types and fragrances of waterpipe, with a relatively affordable price. The recent upsurge in waterpipe smoking among females in Arab countries emphasizes the need to identify and address the gaps in tobacco regulations along with focused interventions targeted to this specific population^4^.

Waterpipe smoking has considerable short- and long-term harmful health effects. Its short-term health issues may include headache, nausea, and dizziness, among others1. Long-term health effects include respiratory and pulmonary diseases as well as cardiovascular complications^45-47^. As seen in our findings, cough was the third most prevalent health symptom among males and older age categories specifically 35–44 years and 45–54 years. It is apparent that waterpipe smoking poses a significant public health risk. One of the alternative ways to prevent and reduce smoking prevalence among the community is to empower health professionals by training them to identify waterpipe smokers, educate them about potential harmful effects, and refer them to cessation services. Fortunately, according to a recent study conducted among governmental healthcare workers in Qatar, about 60% of them routinely promoted tobacco cessation interventions, thus being effective in helping users to quit^28^.

Males more than females were using five or more waterpipes per week (35.6% vs 24.1%). Likewise, about half of Qataris (44.1%) smoked five or more waterpipes per week, these findings raise concerns regarding the dependence of waterpipe use among Qataris, which can lead to addiction and hazardous effects on health^17-19^.

The age of initiation for waterpipe smoking is much dependent on the age range of the sample studied and it is unlike cigarettes, where most initiation usually takes place before the age of 18 years^2^. According to the Qatar 2013 GATS survey, among people aged ≥15 years, approximately 11% of waterpipe smokers started waterpipe smoking before the age of 18 years^35^. However, more than half of Qataris (52.1%) started smoking waterpipe before the age of 18 years. Our sample included individuals ≥18 years making it hard to strictly compare the two findings, but it still provides an insight. Knowing that most long-term smokers start smoking from the age of 18 to 25 years and that young adulthood is the time when individuals are most susceptible to starting tobacco use, it is recommended that the legal age to buy tobacco is raised to 21 years and higher taxation is introduced on waterpipe tobacco to decrease its affordability like cigarette taxation^48^ and waterpipe bottles labelled with health warnings^27^. In 2018, the Ministry of Finance increased the minimum import duty from 100 to 200 Qatari Riyals per 1000 cigarettes. The minimum import duty on waterpipe tobacco was 6 Qatari Riyals per kilogram in 2014, this was increased to 12 Qatari Riyals per kilogram in 2016^49^ and to 24 Qatari Riyals in 2018, because of tobacco taxation set at 100%^34^. Although government efforts are underway, further urgent action can lead to improving the situation.

We found that 83.3% of waterpipe smokers reported that waterpipe smoking is harmful, however 39.3% of the sample considered that waterpipe smoking is less harmful than cigarette smoking. In a systematic review, a large number of studies found that most people^10^ perceived waterpipe smoking as less harmful than cigarette smoking and more socially acceptable than cigarette smoking in general. This is consistent with the findings of previous studies in Qatar reporting that waterpipe smokers perceive waterpipe to be safer than cigarettes or have insufficient knowledge about its effects^9,50^. For instance, in our study only 42.2% of waterpipe smokers agreed that smoking tobacco causes stroke and only 69.2% reported that it causes heart attack. Given the health risks of waterpipe smoking, health campaigns should be delivered to the public to correct misconceptions that contribute toward a reduced perception of harm about tobacco use in general and waterpipe smoking.

In our sample, 75.5% believed that smokers can stop smoking if they want to. However, evidence suggests the opposite, by confirming that waterpipe smokers have difficulty in quitting^19,51^. Among a sample of 268 waterpipe smokers, 86.5% believed they could quit waterpipe at any time, however 60% of the participants had been unsuccessful in their previous attempts^52^. In another study, 47.5% of the participants were very confident that they could quit waterpipe smoking at any time, however they did not have the intention to quit^9^. Surprisingly, in our study females more than males were recommending waterpipe to their friends or to other people. This also highlights the association between the positive attitude toward smoking and waterpipe use^4,10^. Urgently, mass media and awareness campaigns are needed to address the addictive and deleterious health consequences of waterpipe smoking to improve knowledge and to change attitudes towards waterpipe smoking since it is becoming a socially normative behavior as part of leisure events among friends and families^9,51^.

### Strengths and limitations

The major strength of this study was the use of data from a population-based study with a large sample size and a high response rate. Any form of tobacco use is a sensitive topic that many are reluctant to talk about, however the use of self-administered questionnaires helped resolve this issue. One limitation of this study is its focus on individuals aged ≥18 years attending governmental universities and working institutions which might limit the generalizability of these findings to other populations. Due to not adjusting for clusters, it is possible that bias may affect the magnitude and direction of the results.

## CONCLUSIONS

Waterpipe smoking is considerably high in Qatar (8.3%). Waterpipe use should receive special consideration in Qatar’s overall tobacco control program. The current situation in Qatar warrants further implementation of effective strategies to reduce waterpipe smoking with dual and multiple usage of other tobacco products. These strategies could include the introduction of more stringent regulations and legislations specific to waterpipe advertising and marketing, educating the community about its health risks, and designing tobacco control programs for women and for poly-tobacco product users. Compared to earlier years, to date Qatar has been more comprehensively implementing FCTC measures. However, dissemination and implementation of waterpipe preventive measures and regulations deserve due attention by decision makers in the country.

## Data Availability

The data supporting this research are available from the authors on reasonable request.
